# Small cell lung carcinoma presenting initially with recurrent pneumothoraces: a case report

**DOI:** 10.1186/s13019-024-02857-x

**Published:** 2024-06-21

**Authors:** John Buchanan, Mohamed Shatila, Ashvini Menon, Akshay J. Patel

**Affiliations:** 1 University of Birmingham Medical School, Edgbaston, Birmingham, B15 2TT West Midlands UK; 2 Department of Thoracic Surgery, Queen Elizabeth Hospital, University Hospitals Birmingham, UHB Hospitals NHS Foundation Trust, Mindelsohn Way, Edgbaston, Birmingham, B15 2TH England, UK; 3 Department of Cardiothoracic Surgery, Faculty of Medicine, Alexandria University Hospital, Alexandria, Egypt; 4 Institute of Immunology and Immunotherapy, University of Birmingham, Vincent Drive, Edgbaston, B15 2TT UK

**Keywords:** Small cell lung cancer, Chronic obstructive pulmonary disease, Video-assisted thoracoscopic surgery, Pneumothorax

## Abstract

**Background:**

Pneumothorax is a non-physiological collection of air in the pleural space. Pneumothoraces can be broadly divided into Primary, Secondary, and Traumatic. Cancer of the lung is a known cause of secondary pneumothorax in both primary and metastatic lesions, however, pneumothorax as the presentation of lung cancer is exceedingly rare. Non-small cell lung carcinoma (NSCLC) has been reported in the literature to present with a pneumothorax, particularly in adeno/squamous cell carcinomas. It is almost completely unheard of for small cell lung carcinoma (SCLC) to present with a pneumothorax.

**Case Presentation:**

We present the case of a 62-year-old male patient, presenting twice in two months with spontaneous pneumothorax. The initial management involved admission and chest drain insertion. The patient has a past medical history of COPD and a significant smoking history. On the second admission, he underwent a video-assisted thoracoscopic (VATS) bullectomy and talc pleurodesis. The pathology report of the resected specimen confirmed SCLC with extensive infiltration. No gross evidence of metastatic spread was present on CT. Due to the R1 resection and significant risk of recurrence, the management plan included four cycles of adjuvant chemotherapy with carboplatin and etoposide, and radiotherapy as a consideration upon completion.

**Conclusions:**

Pneumothorax as the presentation of lung cancer imparts a very poor prognosis, however the reasons for this are largely unknown. Furthermore, the mechanisms underlying spontaneous pneumothorax in lung cancer are also not well understood.

## Introduction

Cancer of the lung is a known cause of spontaneous pneumothorax (SPx), which can occur in primary or metastatic lesions; metastatic diseases such as sarcoma cause SPx more commonly than primary lung cancers [1]. SPx as the first presentation of primary pulmonary carcinoma has been widely reported to occur in the literature in non-small cell lung carcinomas; potentially due to the fact that this category of carcinoma most frequently occurs in peripheral lung tissue (NSCLC) [2–10], however, in our review of the literature, small cell lung carcinoma (SCLC) has only been reported to present with SPx three times previously [3, 11, 12]. SCLC is characterized by its aggressive and rapid growth, early metastatic potential, and poor survival when compared to NSCLCs [13]. In this case report we present, to the best of our knowledge, the fourth reported instance of SCLC presenting initially with SPx.

## Case presentation

Here we present a 62-year-old gentleman with a history of COPD and recurrent episodes of shingles, who is on regular inhalers. He has no history of COVID-19 infection. Our patient was an ex-smoker with a 40-pack year history, fully independent with a performance status of zero.

**Fig. 1:**
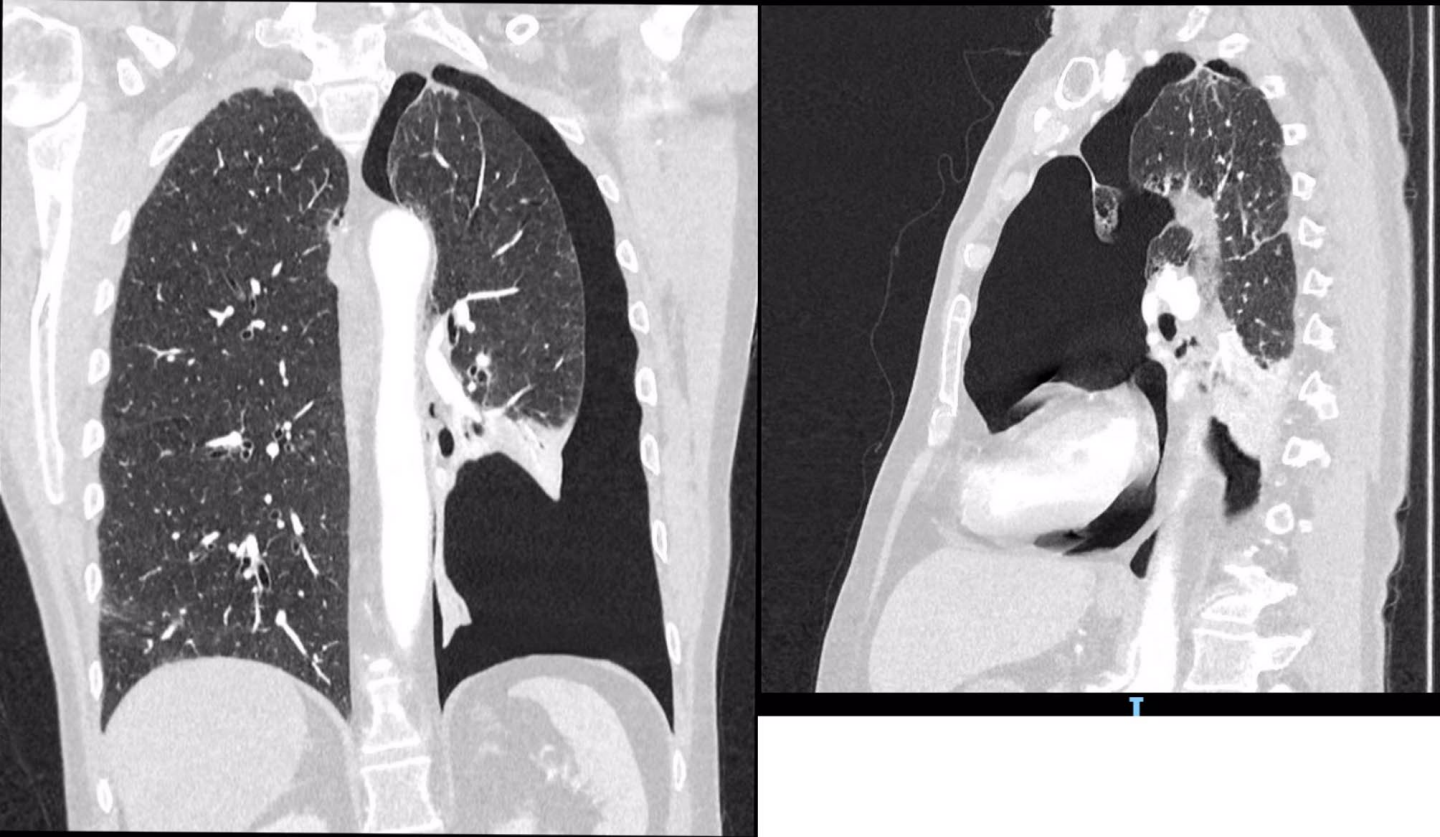
Shows coronal (left) and sagittal (right) views of the CT thorax taken on the patients second admission. A large left-sided pneumothorax can be appreciated

**Fig. 2:**
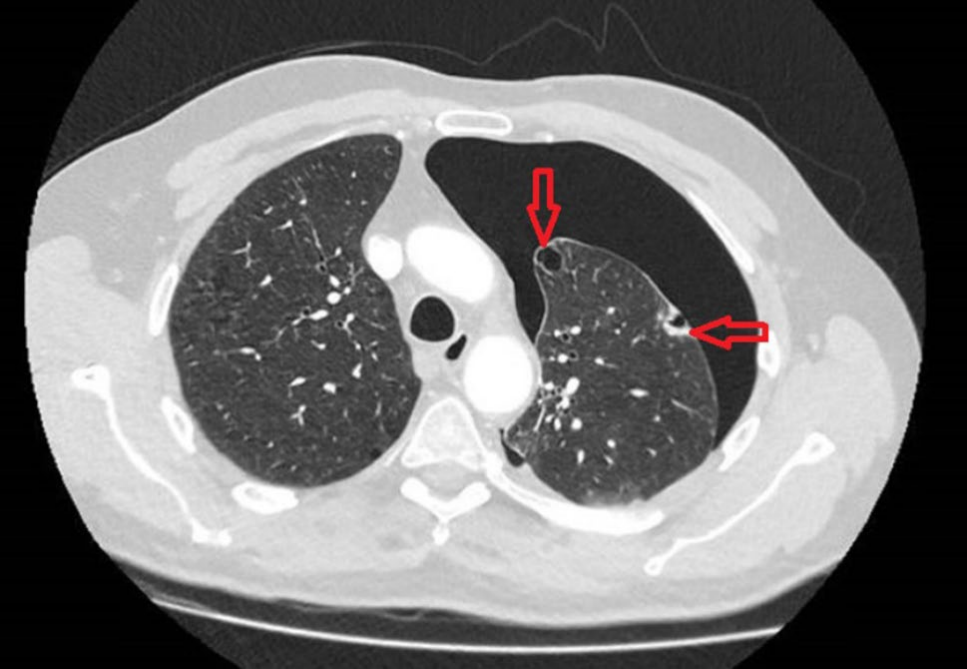
Shows an axial view of the CT taken on the patients second admission. Red arrows indicate significant bullous disease with the furthest right arrow showing the offending portion of the lung

In October 2022, he presented with an acute onset of chest pain and shortness of breath on his left side and was transferred to accident and emergency. Chest x ray showed a large left sided secondary spontaneous pneumothorax. The patient was managed with drain and hospital admission. Air leak settled within 4 days with full lung expansion confirmed on chest x ray and the decision was taken to remove the drain. No talc slurry was used. Two months later he presented with his second episode of symptomatic pneumothorax on the left side which was managed with a 12 Fr drain insertion and another hospital admission. A CT thorax was requested. The CT scan showed a significant left-sided pneumothorax, bullous disease consistent with COPD, and a suspicious thickened left lateral bulla. Figures 1 and 2 show the CT scan on this second presentation.

The persistent air leak for several days warranted referral to the thoracic surgery team for consideration of surgical intervention. The decision was taken to proceed with surgery and the patient was transferred to a thoracic surgery unit.

The patient underwent a left-sided anterior 3-port VATS upper lobe bullectomy and talc pleurodesis. Intraoperatively, bullae were appreciated at the lateral aspect of the upper lobe with adhesions present at the apex. Two bullae were resected from this region using a wedge resection, performed with Endo GIA 40/60 stapler and the apical adhesion was released; the lung tissue was found to be thick, making staple application challenging. Talc pleurodesis was performed using 8 g of talc. A single apical 28 Fr drain was inserted, and the lung fully expanded under vision.

Postoperatively, the drain was connected to an automated suction and kept at -2.5 KpA. POD2 showed no air leak with full lung expansion on x ray and decision was made to home remove drain. Patient was discharged after a satisfactory post-drain removal chest x ray.

The results of the biopsy showed a defect on the pleural surface measuring 15 mm. On slicing, a firm 20 mm cream-coloured area was noted lying directly beneath the defect, measuring 20 mm, and abutting the inked resection margin. Microscopically, it showed a tumour composed of sheets of medium sized cells with enlarged nuclei, little cytoplasm, and a brisk mitotic and apoptotic rate. Immunohistochemistry was positive for synaptophysin with focal TTF1 and chromogranin A staining. The upper lobe wedge showed extensive infiltration with small cell carcinoma, and a diagnosis of limited-stage small cell carcinoma was made The second wedge, also sent for histological analysis, showed only emphysema and fibroelastosis, there was no background evidence of interstitial lung disease.

**Fig. 3:**
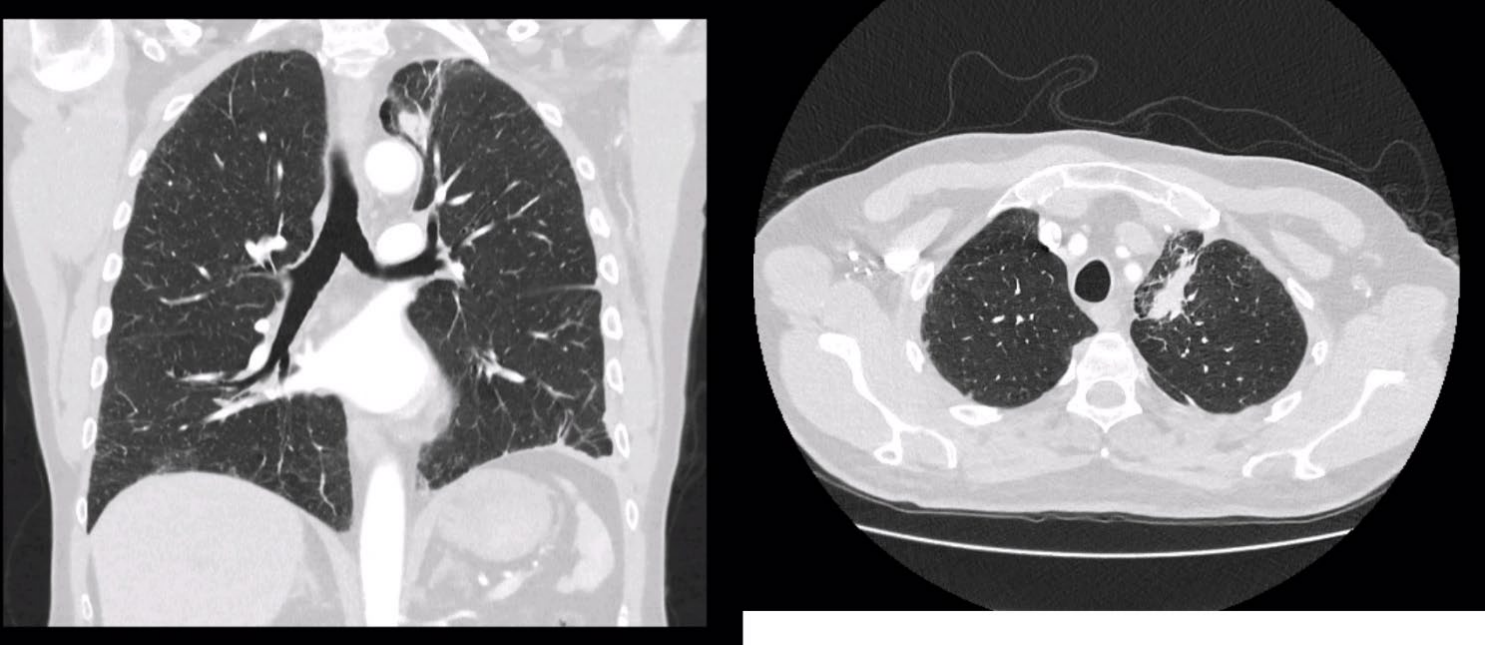
Shows coronal (left) and sagittal (right) views of the CT thorax taken post-operatively. Post-surgical changes can be appreciated in the left lung with no evidence of tumour

Results of the histology were discussed with the patient and in our lung MDT. Urgent CT TAP, PET scan and an MRI brain were requested for radiological staging. MRI head showed no evidence of intracranial metastasis. Completion lobectomy and adjuvant chemotherapy were discussed at the MDT; the patient was referred to the oncology team for adjuvant chemotherapy. He received 4 cycles of carboplatin and etoposide along with prophylactic cranial irradiation. Follow up CT thorax showed only post-surgical changes with no evidence of tumor (Fig. 3). Patient is currently doing well, being followed up by the oncology team.

## Comment

A Spontaneous Pneumothorax (SPx) is a pneumothorax occurring due to any non-traumatic aetiology and can be broadly divided into Primary SPx (PSP) and Secondary SPx (SSP). PSP is commonly defined as pneumothorax in the absence of apparent lung pathology whereas SSP is pneumothorax in the presence of underlying lung disease, most commonly COPD [14]. PSP usually occurs in the first three decades of life whereas SSP has a peak incidence in the 7th [14]. The distinction between these broad categories for SPx are debatable and can represent a grey area. It is widely known that bullous disease and emphysematous lung changes can directly cause SSP via rupture of bullae or subpleural blebs. Due to advancements in modern imaging modalities such as CT, emphysematous changes can be appreciated in many of those who present with PSP [15]. In this way, PSP and SSP represent a spectrum of lung disorders causing pneumothorax; from subclinical, to symptomatic and widespread disease. Hence, one can appreciate how these categories can seem arbitrary as the mechanism of pneumothorax is often the same. There is still a clinically useful role for this distinction however, as the management is frequently more difficult, and the prognosis poorer in SSPs when compared to PSPs [16]. As is relevant for this case, SSP is managed acutely with a chest drain and supplementary oxygen; spontaneous resolution is less likely in SSP than PSP and therefore definitive therapies including pleurodesis and pleurectomy are not infrequently required [16].

Invariably, in SPx occurring with lung cancer, the insult is ipsilateral to the pulmonary tumour [5]; there are various proposed theories for this process. The main prevalent ideas in the literature consider the tumour to be causing the SPx; these include the creation of a bronchopleural fistula, the check-valve mechanism and compensatory overdistension, however, it is widely appreciated that these events may instead be in association rather than causation [17]. In this instance, we have coexistent lung cancer and COPD and therefore we cannot rule out a coincidental pneumothorax due to the COPD, unrelated to subsequently identified cancer. Nevertheless, the presence of SCLC at the site of resection (presumed weakened area in the lung parenchyma leading to pneumothorax) is an incredibly rare finding and concurrent malignancy as a potentially causative event in the aetiopathogenesis of pneumothorax should always be considered at the time of surgery in high-risk patients, who are frail, poor-quality parenchyma with an extensive smoking history.

‘Lung cancer associated with cystic airspaces’ has been described in the literature as a rare presentation of lung cancer whereby bullae or ‘cysts’ cause airflow obstruction, leading to reduced pulmonary clearance and the facilitation of carcinogen deposition and accumulation [18, 19]. In this way we could consider the cancer to be a direct result of emphysematous lung changes. It has been shown that this manifestation of lung cancer is associated with both smoking and emphysema, however this association was only investigated in NSCLCs [20]. From the appearance of the CT imaging and histological findings in our case, we propose that this SCLC may represent a bullous or ‘cystic airspace’ associated cancer, whereby a bulla has undergone carcinogenesis, grown, and ruptured; due to its peripheral location, this rupture then led to recurrent pneumothoraces.

## Data Availability

No datasets were generated or analysed during the current study.

## Abbreviations

SCLC: Small Cell Lung Cancer
NSCLC: Non–small Cell Lung Cancer
COPD: Chronic Obstructive Pulmonary Disease
VATS: Video–assisted Thoracoscopic Surgery
CT: Computed Tomography
SPx: Spontaneous Pneumothorax
POD2: Post–operative Day 2
TTF1: Thyroid Transcription Factor 1
MDT: Multidisciplinary Team
TAP: Thorax, Abdomen, Pelvis
PET: Positron Emission Tomography
MRI: Magnetic Resonance Imaging
PSP: Primary Spontaneous Pneumothorax
SSP: Secondary Spontaneous Pneumothorax

## References

[1] Abu Arab W, Ramadan A (2020). Spontaneous pneumothorax associated with primary lung cancer: a retrospective study. Cardiothorac Surg.

[2] Yeung KY, Bonnet JD (1977). Bronchogenic carcinoma presenting as spontaneous pneumothorax: case reports with review of literature. Cancer.

[3] Steinhäuslin CA, Cuttat JF (1985). Spontaneous pneumothorax. A complication of lung cancer?. Chest.

[4] Galbis Caravajal JM, Mafé Madueño JJ, Baschwitz Gómez B, Pérez Carbonell A (2001). Rodríguez Paniagua JM. [Spontaneous pneumothorax as the first sign of pulmonary carcinoma]. Arch Bronconeumol.

[5] Vencevicius V, Cicenas S (2009). Spontaneous pneumothorax as a first sign of pulmonary carcinoma. World J Surg Oncol.

[6] Choi YK, Kim KC (2015). Spontaneous pneumothorax as the first manifestation of lung cancer: two case report. J Thorac Dis.

[7] Mahajan V, Kupferer CF, Van Ordstrand HS, Pneumothorax (1975). A rare manifestation of primary lung cancer. Chest.

[8] Lundgren R, Stjernberg N. Spontaneous pneumothorax as first symptom in bronchial carcinoma. Acta Med Scand. 1980;207(4):329 – 30. 10.1111/j.0954-6820.1980.tb09730.x. PMID: 7386226. 10.1111/j.0954-6820.1980.tb09730.x 7386226

[9] Galbis Caravajal JM, Mafé Madueño JJ, Baschwitz Gómez B, Pérez Carbonell A, Rodríguez Paniagua JM (2001). Neumotórax espontáneo como primera manifestación de un carcinoma pulmonar [Spontaneous pneumothorax as the first sign of pulmonary carcinoma]. Arch Bronconeumol.

[10] Okada D, Koizumi K, Haraguchi S (2002). Pneumothorax manifesting primary lung cancer. Jpn J Thorac Cardiovasc Surg.

[11] Dines DE, Cortese DA, Brennan MD, Hahn RG, Payne WS. Malignant pulmonary neoplasms predisposing to spontaneous pneumothorax. Mayo Clin Proc. 1973;48(8):541-4. PMID: 4352472. 4352472

[12] Ayres JG, Pitcher DW, Rees PJ (1980). Pneumothorax associated with primary bronchial carcinoma. Br J Dis Chest.

[13] Thai AA, Solomon BJ, Sequist LV, Gainor JF, Heist RS. Lung Cancer. The Lancet. 202;398(10299):535–554. 10.1016/S0140-6736(21)00312-3. 10.1016/S0140-6736(21)00312-3 34273294

[14] Onuki T, Ueda S, Yamaoka M, Sekiya Y, Yamada H, Kawakami N, Araki Y, Wakai Y, Saito K, Inagaki M, Matsumiya N (2017). Primary and secondary spontaneous pneumothorax: prevalence, clinical features, and In-Hospital mortality. Can Respir J.

[15] Feller-Kopman D, Light R, Pleural Disease. N Engl J Med. 2018;378(8):740–751. 10.1056/NEJMra1403503. PMID: 29466146. 10.1056/NEJMra1403503 29466146

[16] MacDuff A, Arnold A, Harvey J (2010). Management of spontaneous pneumothorax: British thoracic society pleural disease guideline 2010. Thorax.

[17] O’Connor BM, Ziegler P, Spaulding MB. Spontaneous pneumothorax in small cell lung cancer. Chest. 1992;102(2):628-9. 10.1378/chest.102.2.628. PMID: 1322814. 10.1378/chest.102.2.628 1322814

[18] Goldstein MJ, Snider GL, Liberson M, Poske RM. Bronchogenic carcinoma and giant bullous disease. Am Rev Respir Dis. 1968;97(6):1062-70. 10.1164/arrd.1968.97.6P1.1062. PMID: 5658877. 10.1164/arrd.1968.97.6P1.1062 5658877

[19] Snoeckx A, Reyntiens P, Carp L, Spinhoven MJ, El Addouli H, Van Hoyweghen A, Nicolay S, Van Schil PE, Pauwels P, van Meerbeeck JP, Parizel PM (2019). Diagnostic and clinical features of lung cancer associated with cystic airspaces. J Thorac Dis.

[20] Fintelmann FJ, Brinkmann JK, Jeck WR, Troschel FM, Digumarthy SR, Mino-Kenudson M, Shepard JO. Lung Cancers Associated With Cystic Airspaces: Natural History, Pathologic Correlation, and Mutational Analysis. J Thorac Imaging. 2017;32(3):176–188. 10.1097/RTI.0000000000000265. PMID: 28338535. 10.1097/RTI.0000000000000265 28338535

